# The Role of Hydrophobicity and Surface Receptors at Hyphae of *Lyophyllum* sp. Strain Karsten in the Interaction with *Burkholderia terrae* BS001 – Implications for Interactions in Soil

**DOI:** 10.3389/fmicb.2016.01689

**Published:** 2016-10-27

**Authors:** Taissa Vila, Rashid Nazir, Sonia Rozental, Giulia M. P. dos Santos, Renata O. R. Calixto, Eliana Barreto-Bergter, Lukas Y. Wick, Jan Dirk van Elsas

**Affiliations:** ^1^Laboratório de Biologia Celular de Fungos, Instituto de Biofísica Carlos Chagas Filho, Universidade Federal do Rio de JaneiroRio de Janeiro, Brazil; ^2^Department of Microbial Ecology, Groningen Institute for Evolutionary Life Sciences, University of GroningenGroningen, Netherlands; ^3^Department of Environmental Sciences, COMSATS Institute of Information TechnologyAbbottabad, Pakistan; ^4^Laboratório de Química Biológica de Microrganismos, Instituto de Microbiologia Paulo de Goes, Universidade Federal do Rio de JaneiroRio de Janeiro, Brazil; ^5^Department of Environmental Microbiology, UFZ - Helmholtz Centre for Environmental ResearchLeipzig, Germany

**Keywords:** *Burkholderia terrae*, *Lyophyllum* sp. Karsten, fungal-bacterial association, cerebroside, CMH, soil fungi

## Abstract

The soil bacterium *Burkholderia terrae* strain BS001 can interact with varying soil fungi, using mechanisms that range from the utilization of carbon/energy sources such as glycerol to the ability to reach novel territories in soil via co-migration with growing fungal mycelia. Here, we investigate the intrinsic properties of the *B. terrae* BS001 interaction with the basidiomycetous soil fungus *Lyophyllum* sp. strain Karsten. In some experiments, the ascomycetous *Trichoderma asperellum* 302 was also used. The hyphae of *Lyophyllum* sp. strain Karsten were largely hydrophilic on water-containing media versus hydrophobic when aerial, as evidenced by contact angle analyses (CA). Co-migration of *B. terrae* strain BS001 cells with the hyphae of the two fungi occurred preferentially along the - presumably hydrophilic - soil-dwelling hyphae, whereas aerial hyphae did not allow efficient migration, due to reduced thickness of their surrounding mucous films. Moreover, the cell numbers over the length of the hyphae in soil showed an uneven distribution, i.e., the CFU numbers increased from minima at the inoculation point to maximal numbers in the middle of the extended hyphae, then decreasing toward the terminal side. Microscopic analyses of the strain BS001 associations with the *Lyophyllum* sp. strain Karsten hyphae in the microcosms confirmed the presence of *B. terrae* BS001 cells on the mucous matter that was present at the hyphal surfaces of the fungi used. Cell agglomerates were found to accumulate at defined sites on the hyphal surfaces, which were coined ‘fungal-interactive’ hot spots. Evidence was further obtained for the contention that receptors for a physical bacterium-fungus interaction occur at the *Lyophyllum* sp. strain Karsten hyphal surface, in which the specific glycosphingolipid ceramide monohexoside (CMH) plays an important role. Thus, bacterial adherence may be mediated by heterogeneously distributed fungal-specific receptors, implying the CMH moieties. This study sheds light on the physical aspects of the *B. terrae* BS001 – *Lyophyllum* sp. strain Karsten interaction, highlighting heterogeneity along the hyphae with respect to hydrophobicity and the presence of potential anchoring sites.

## Introduction

Next to bacteria, the fungi in soil are responsible for key ecosystem functions (De Boer et al., 2005). Several soil fungi show interactions with bacteria, of mutualistic, commensalistic, and/or antagonistic nature (De Boer et al., 2005; Mille–Lindblom et al., 2006; Nazir et al., 2010; Frey-Klett et al., 2011; Haq et al., 2014). For example, *Amanita muscaria* counters the antibiotics produced by associated *Streptomyces* sp. AcH505 by releasing organic acids (Riedlinger et al., 2006). *A*sp*ergillus niger* is affected by collimomycins produced by *Collimonas fungivorans* associated with it (Fritsche et al., 2014). Clearly, soil fungi often ‘tolerate’ bacterial associates, even slowing their growth rate, to allow co–existence (Mille–Lindblom et al., 2006). Nazir postulated that interactions can thus be mutualistic (Nazir, 2012).

In previous work in our laboratory, a predominance of different *Burkholderia* species, in particular *Burkholderia terrae*, was found in the soil underneath the foot of *Laccaria proxima* fruiting bodies, denoted the mycosphere (Warmink and van Elsas, 2008). Subsequently, similar *Burkholderia* types turned out to be enriched in soil that is colonized by a closely related fungus, the saprotrophic fungus *Lyophyllum* sp. strain Karsten (Warmink and van Elsas, 2009). Thus, *B. terrae* strains BS001 (interactive with *Lyophyllum* sp. strain Karsten; Warmink and van Elsas, 2009; Warmink et al., 2011) and BS110 (interactive with *Laccaria proxima;* Warmink and van Elsas, 2008) were identified as typical mycosphere dwellers. In later work, co-migration of strain BS001 along the hyphae of a suite of soil fungi, including *Trichoderma asperellum* 302, was found (Nazir et al., 2014). This co-migration capacity was spread across several species related to *B. terrae* (Nazir et al., 2012), and so this particular subgroup within the genus *Burkholderia* might be denoted as a group of ‘potentially fungal-interactive’ soil bacteria.

The association of *B. terrae* BS001 with fungal surfaces is likely to have a physical component. For the establishment of a successful interactive pair, this presumably involves a recognition phase, followed by a, possibly multifaceted, physical interaction between bacterial and fungal surface components. From the bacterial side, a role for the type 3 secretion system (T3SS) and/or for type 4 pili has been proposed (Yang et al., 2016). However, there has so far been no clue as to the existence of any dedicated ‘receptor’ site at the fungal cell surface, even though Haq et al. (2016) recently found evidence for the presence of ceramide monohexosides (CMH) as molecular ‘rafts’ in the cell surface of *Trichoderma asperellum* 302. In previous studies, we have reported the finding of biofilm-like structures (cell agglomerates) consisting of *B. terrae* BS001 cells at the hyphae of *Lyophyllum* sp. strain Karsten, as well as other fungi (Nazir et al., 2014). However, it is so far unknown how and to what extent *B. terrae* BS001 colonizes the fungal hyphae, as related to the physical and chemical parameters that characterize the hyphal surface. Here, the molecular structure, in particular the role of CMH rafts, and the surface hydrophobicity of the hyphal cell walls are key aspects to be addressed.

In this study, we thus extend the previous work on *B. terrae* BS001 migration along the hyphal networks of *Lyophyllum* sp. strain Karsten (Warmink et al., 2011) by addressing the following research questions: (i) Is the migration of *B. terrae* BS001 along the hyphae of soil fungi dependent on the surface hydrophobicity of the latter? (ii) To what extent and in what fashion does *B. terrae* BS001 colonize the fungal hyphae? (iii) Is there a specific surface receptor on the fungal hyphae that may serve as a cell-binding (anchoring) site? Using [mainly] the reference system *B. terrae* BS001 – *Lyophyllum* sp. strain Karsten, we performed interaction experiments both in soil microcosms and *in vitro*, followed by observations by (electron) microscopy.

## Materials and Methods

### Strains and Culture Conditions

The fungal strains used in this study, i.e., the basidiomycete *Lyophyllum* sp. strain Karsten (DSM2979) and the ascomycete *Trichoderma asperellum* 302, were routinely grown on oat flake agar (OFA) plates, prepared with 30 g of oat flakes as obtained from a grocery shop and 15 g of agar (Duchefa, Haarlem, The Netherlands) in milliQ water to 1 l, and sterilized at 121°C for 21 min. Once every 4 weeks, the fungal cultures were transferred to fresh OFA plates for maintenance. Prior to each experiment, fungal pieces were transferred to Erlenmeyer flasks with 200 *ml* of fresh potato dextrose (PD) broth and allowed to grow for 7 days (room temperature, with shaking).

Two *B. terrae* strains were used, i.e., the wild-type strain BS001 and its gfp-modified derivative BS001-GFP (tagged previously and described in Nazir et al., 2014). The strains were kept in 20% glycerol stored at -80°C. Prior to each experiment, bacteria were grown for 72 h in 5 ml of LB broth at 28°C.

### Determination of Fungal Growth

*Lyophyllum* sp. strain Karsten mycelium grown for 7 days in PD broth was transferred to sterile Falcon tubes (50 ml) using a 50 ml glass pipette, and vortexed vigorously to homogenize. The suspension was then centrifuged (3000 RPM) in a swinging bucket rotor centrifuge for 10 min. The pellet containing fungal propagules was washed three times with 0.01 M phosphate buffered saline (PBS; pH 7.2). After the last wash, the pellet was resuspended in fresh PD broth. An aliquot (500 μL) of this standardized fungal suspension (assuring that similar inoculum sizes were used to start the culture each time the experiment was carried out) was added to each well of a sterile 24-well microplate (TPP, Techno Plastic Products, Switzerland) and allowed to grow for 3, 24, 48 and 72 h, at room temperature, in static conditions. At each time point, the resulting fungal biomass was quantified using a crystal violet (CV) assay, as described earlier (Silva et al., 2009). First, biofilms formed in 24-well microtiter plates were fixed with 100 μL of methanol for 15 min at room temperature. Then, supernatants were removed and plates air-dried. Next, 500 μL of CV solution (0.02%, Sigma-Aldrich; USA) was added to each well and plates were kept for 20 min. Excess dye was then removed and plates were washed three times with distilled water. Lastly, 400 μl of 33% acetic acid (Merck Millipore, USA) was added and plates kept for 5 min. Then, 100 μL from each well was transferred to a 96-well microtiter plate (TPP, Techno Plastic Products; Switzerland) and absorbance at 590 nm quantified by a plate reader (Spectra-MAX 340 Tunable Microplate Reader, Molecular Devices Ltd.; USA).

### Contact Angle (CA) Analysis

Water contact angles (CA) of aerial mycelia of *Lyophyllum* sp. strain Karsten were measured after growth on cellulose acetate membrane filters (diameters of 25 mm; pore size 0.45 μm, type OE 67, Schleicher and Schuell, Dassel, Germany) overlying either malt extract agar (MA) or OFA as previously described (Smits et al., 2003). Shortly, 3 μL of fungal mycelium suspension was placed as the inoculum on top of the center of each filter and the fungus allowed to fully overgrow the filter (ca. 14-20 days). Mycelium-covered filters were removed from the plate and washed three times under suction with 20 ml 10 mM KNO_3_. Afterwards, the filters were mounted on a microscopy glass slide, air-dried for either 2, 4 or 6 h at room temperature and the CA of the upper side of the mycelia derived from the CA of >8 water droplets placed on the mycelia of a least two independent growth experiments using a goniometric eye piece (Krüss GmbH, Hamburg, Germany). As hyphal surfaces are known to vary their CA in response to humidity, growth medium and physiological status (Smits et al., 2003), two further experiments were performed. In order to simulate the effect of humidity and nutrients on the CA, mycelia-covered filters were submerged three days after inoculation in a thin sterile physiological 0.9% NaCl solution (ca. 8 ml per agar plate) in separate experiments. Although physiological NaCl solution is primarily free of nutrients, it will dissolve water-soluble compounds from the underlying MA or OFA and hence provide nutrients to the hyphae. After ca. 14 days, the overgrown filters were harvested, washed as described above and allowed to dry in air either for 4 or 6 h before CA analysis. Secondly, in order to assess the effects of possible hydrophobin formation on the CA during drying, identical experiments were performed, yet the filters exposed (for 10 min) to 15 ml of 0.2% cycloheximide (CH) prior to drying for 4 and 6 h and subsequent CA analysis, respectively. CH was used during drying as a eukaryotic protein synthesis inhibitor to allow assessment of hydrophobin-related impacts on the CAs.

The CA of *B. terrae* BS001 cells was determined as described earlier (Wick et al., 2002), using early stationary-phase cells growing in minimal medium in the presence of 1 g/l of glucose.

### Microscopic Assays of Bacterial-Fungal Interactions: Growth Conditions

Fungal biomass (*Lyophyllum* sp. strain Karsten) grown for 7 days was prepared as described above. An aliquot of 500 μL was added to each well of a 24-well microplate (TPP, Techno Plastic Products, Switzerland) containing a round glass slide or to a glass-bottom dish (CELLview^TM^, Greiner Bio-One, Germany) and allowed to adhere for 90 min at room temperature under static conditions. Following this, the supernatant was removed and a standardized suspension of strain BS001-GFP cells (10^7^ CFU/*ml*) was added to each well (500 μL). The plate was incubated for 3, 5 or 24 h at room temperature under static conditions. Following incubation, the supernatant was removed and the biomass adhering to the microplate (or to the glass slide) was washed with PBS for three times to remove any loosely attached bacteria. Finally, the systems were prepared for microscopic analyses.

### Confocal Laser Scanning Microscopy (CLSM): Sample Preparation and Analysis

The biomass adhering to the glass-bottom dish wells was fixed in 4% formaldehyde in 0.1 M phosphate buffer for one hour at room temperature. Next, the samples were treated with Calcofluor white M2R (0.05% v/v in distilled water; Sigma-Aldrich, USA) to stain fungal cell walls (10 min, room temperature). Then, samples were washed in PBS (at least six times) and submerged in PBS for analysis by CLSM (Leica TCS SPE, Leica Mycrosystems, Germany). CLSM images were analyzed for fluorescence intensity using the ImageJ software (NIH, USA). The biomass (Z-stack) was virtually divided in four parts: top, middle, deep and bottom. At least 20 confocal planes were analyzed in each image (*n* = 2, Z-stacks, five planes for each virtual part) and, in each plane, 40-50 hyphae or hyphal areas were selected for fluorescence intensity quantification. Each area analyzed generates a fluorescence intensity graph containing all three dyes used, allowing comparison between them.

### Electron Microscopy: Sample Preparation and Analysis

Glass slides containing the strain BS001 – *Lyophyllum* sp. strain Karsten association, as well as samples of each species alone, were processed for scanning electron microscopy (SEM). *Lyophyllum* sp. strain Karsten alone was also processed for transmission electron microscopy (TEM). Briefly, samples were washed in 0.01 M PBS, pH 7.2 (at least twice, to remove all non-adhered bacteria from the hyphae) and fixed in 2.5% glutaraldehyde and 4% formaldehyde, in 0.1 M cacodylate buffer, for 1 h at room temperature. Subsequently, samples were washed in the same buffer, post-fixed in 1% osmium tetroxide (OsO_4_) and 1.25% potassium ferrocyanide for 2 h and then dehydrated in a series of increasing ethanol concentrations (30%, 50%, 70%, 90%, 100%, and ultra-dry ethanol) for 30 min at each dehydration step. For SEM, samples were critical-point-dried in CO_2_, coated with gold and observed under a FEI Quanta 250 scanning electron microscope (FEI, The Netherlands). For TEM, dehydrated samples were slowly embedded in Spurr resin (Electron Microscopy Sciences - EMS, USA). Ultrathin sections of the samples were stained with uranyl acetate and lead citrate, and images were obtained in a FEI Spirit Transmission electron microscope (FEI, The Netherlands). All images were processed with Adobe Photoshop CS5 (Adobe System Co., USA).

### Effect of Anti-CMH Antibodies on Bacterial-Fungal Interactions

*Lyophyllum* sp. strain Karsten grown for 7 days was prepared as described in the Section “Determination of Fungal Growth”. An aliquot of 500 μL of fungal cells in water was added to each well of a 4-well glass-bottom dish (CELLview^TM^, Greiner Bio-One, Germany) and allowed to adhere for 90 min at room temperature in static conditions. The supernatant was then removed and fungal biomass was incubated with 2% BSA solution (blocking solution) for 1 h. After incubation, the biomass was washed three times with sterile PBS and treated with anti-CMH antibody at 200 μg/ml for 1 h. After that, the biomass was washed four times with sterile PBS and incubated with anti-mouse secondary antibody IgG conjugated to the fluorochrome Alexafluor 546 for 1 h. At this point, the complex “CMH antibody-anti-IgG conjugate” was blocking all CMH moieties at the fungal cell wall. Then, after several washes with sterile PBS, a standardized suspension of *B. terrae* strain BS001-GFP, prepared in sterile water (10^7^ CFU/*ml*), was added to three wells of the 4-well glass-bottom dish (the last well did not receive bacteria to serve as control). The system was incubated for 3h (initial interaction) or 24 h (late interaction) at room temperature (∼ 25°C) in static conditions, to allow the bacterial cells to interact with the fungal surface. Following incubation, the supernatant was removed, and the biomass washed with sterile PBS at least three times to remove any remaining non-adherent bacteria. Finally, samples were taken for subsequent analyses.

### Extraction and Purification of Glucosylceramides (GlcCer) from *Lyophyllum* sp. Strain Karsten

*Lyophyllum* sp. strain *Karsten* cells were extracted at room temperature using chloroform/methanol at 2:1 and 1:2 (v/v) ratio’s. Extracts were combined and concentrated in vacuum and crude lipid fractions partitioned (Folch et al., 1957). The lipids recovered from the Folch lower layer were fractionated on a silica gel column and eluted with chloroform, acetone and methanol. The methanol fraction, containing glycosphingolipids, was further purified by silica gel column chromatography. Columns were sequentially eluted with chloroform/methanol containing increasing concentrations of methanol (95:5, 9:1, 8:2, 7:3, and 1:1 v/v) and, finally, with methanol. Fractions were analyzed on thin layer chromatography (TLC) plates developed with CHCl_3_: MeOH: NH_4_OH (2 mol/l) (40:10:1 v/v/v) and visualized with iodine vapor, as well as by spraying with orcinol-sulfuric acid (Svennerholm, 1956). The chloroform/methanol 8:2 (v/v) and 7:3 fractions were further purified by silica gel chromatography, using the same elution system to obtain a purified glycosphingolipid fraction. The purified fraction was then analyzed by electrospray ionization mass spectrometry (ESI-MS) in the positive ionization mode. See Supplementary Figure S1.

### ESI-MS Analysis of *Lyophyllum* sp. Strain Karsten GlcCer

Samples were prepared in CH_3_OH at 1 mg/ml, then diluted to 0.1 mg/ml in CH_3_OH:H_2_O (7:3 v/v) containing 1 mM LiCl, as in Rollin-Pinheiro et al. (2014). ESI-MS analysis was carried out in a model Quattro-LC (Waters, Milford, MA, USA) with a triple-quadrupole mass analyzer, operating at atmospheric pressure ionization (API), assisted by a syringe pump (KD Scientific) for sample infusion. Nitrogen was used as the nebulizing and desolvation gas and ionization energies were 80 V (cone) and 2.5 kV (capillary), when operating in the positive ionization mode. Second-stage tandem MS was obtained by collision induced dissociation mass spectrometry (CID-MS) using argon as the collision gas and collision energy ranging between 35 and 60 eV.

### Interaction of *B. terrae* BS001 with Growing Fungal Hyphae in Soil Microcosms

Suspensions of strain BS001 were grown overnight in 5 ml of Luria-Bertani (LB) medium (pH 7.0; Sigma-Aldrich, Haarlem, Netherlands) at 23°C, with shaking. The cells were spun down for 5 min at 5,000 × *g*, washed, and re-suspended in 1 ml of sterile saline (0.85% w/vol NaCl). This procedure was repeated twice. Final cell suspensions adjusted to contain about 10^7^ cells ml^-1^, as evidenced using dilution plating on R2A agar, were used (50 μl) directly for inoculation of soil for the interaction assays.

The microcosms used in this study have been described and validated in several previous studies (Warmink and van Elsas, 2009; Nazir et al., 2012, 2013, 2014). Briefly, they consisted of three-compartment Petri dishes (Greiner Bio one, Frickenhausen, Germany), of which one served as a fungal source and two had sterilized soil. The systems were inoculated, in replicates, with *Lyophyllum* sp. strain Karsten, next to the other selected fungus, *Trichoderma asperellum* 302 (Nazir et al., 2014), on the OFA medium, and incubated at 28°C. Thereby, colonization of the OFA compartment plus about 1 mm of the soil took place prior to introduction of the bacterial inocula. Control plates had no fungal inoculum. The washed suspensions (50 μl) of *B. terrae* strain BS001 cells were placed evenly in one 3-mm-wide streak in the soil compartments adjacent to the hyphal growth fronts. Control treatments consisted of the addition of BS001 cells in a similar streak to microcosms without fungal mycelium, and of sterile water added to the fungal-plus microcosms in a similar streak. The systems were incubated at 23°C (keeping soil moisture content even, at about 70% of soil water holding capacity) and spatially explicit sampling was performed appropriately. Thus samples of about 100 mg were punched out (using an auger; 4 mm diameter) at different sites within the soil compartments. Triplicate samples were used for the further analyses by different methods, including dilution plating according to Nazir et al. (2013) and microscopy. For this, the aboveground and soil parts were split, after which fungal biomass was determined in each compartment using hyphal separation [for soil] and microscopic quantification of hyphal length (Zhang and Zhang, 1994; Shen et al., 2016). The microscopy further included the use of sterile cover slips in the soil microcosms at a point distant from the fungal and bacterial inocula. When the fungal migration front had encroached upon the cover slips, these were carefully removed from the microcosms and analyzed under the microscope. For the analyses, a Zeiss microscope (Zeiss, Jena, Germany) with phase contrast was used.

### Data Analysis

All experiments were performed in triplicate per treatment. At each time point, the CFU data were log-transformed, after which average values and standard deviations (shown between brackets in the text and/or as error bars in the figures) were calculated. Comparisons were made by statistical tests (Student’s *t*-test, ANOVA) using the SPSS package (SPSS, IBM, Statistics 1.8 for Windows), and data are reported as being significant at *P* < 0.05.

## Results

### Effect of Growth Conditions on the Hydrophobicity of Mycelia of *Lyophyllum* sp. Strain Karsten

The CA of small water droplets on the mycelial mats of *Lyophyllum* sp. strain Karsten growing under conditions of varying humidity and substrate availability were quantified. Mycelia exposed to air (i.e., to dry conditions, as occurring in aboveground mycelium) were highly hydrophobic, i.e., 125 ± 5° (MA) and 126 ± 2° (OFM). This was independent of the growth substrate and drying time of the filters (**Table 1**). Mycelia growing submerged in 0.9% NaCl solution (as a mimic for soil pore water) were significantly less hydrophobic, exhibiting CAs of 64 ± 20° (OFM) and 32 ± 6° (MA) (**Table 1**). Increasing the drying period to 6 h changed the CA to 61° on MA, and 56° on OFM, respectively, indicating the possible formation of hydrophobins at the hyphal surfaces. In order to assess hydrophobin-related impacts on the CAs, during drying we prepared mycelia that were treated by a known inhibitor of eukaryotic protein biosynthesis, CH. The CH -treated cells exhibited clearly reduced CA of 14 ± 1° (MA) and 44 ± 11° (OFM), that were virtually independent of the drying time (**Table 1**).

**Table 1:** Effects of growth conditions and drying time on the contact angles (CA) of mycelia of *Lyophyllum* sp. strain Karsten.

Time^1^	CA (degrees)					
	Oat flake agar (OFA)			Malt agar (MA)		
	Wet^2^	Wet+C^3^	Air^4^	Wet^2^	Wet+C^3^	Air^4^
4 h	64 ± 20	44 ± 11	126 ± 2	32 ± 6	14 ± 1	125 ± 5
6 h	56 ± 6	38 ± 1	n.a.	61 ± 6	19 ± 1	121 ± 3

*^1^Drying time of filter; ^2^Growth of mycelia submersed in 0.9% NaCl solution; ^3^Growth of mycelia submersed in 0.9% NaCl solution and filters treated with 0.2% cycloheximide (CH)[C] prior to air drying; ^4^mycelia growing exposed to air.*

### *B. terrae* BS001 Interactions with Fungal Hyphae in Soil Microcosms

We previously reported (Nazir et al., 2012, 2014) that *B. terrae* BS001 migrates along with developing hyphae of *Lyophyllum* sp. strain Karsten and other fungi through soil, establishing varying population densities at the migration front. Here, we evaluated the type of hyphae that may facilitate the bacterial co-migration and also the distribution of bacterial cells over the mycelial network.

#### Spatial Distribution of *B. terrae* BS001 Cells

**FIGURE 1:**
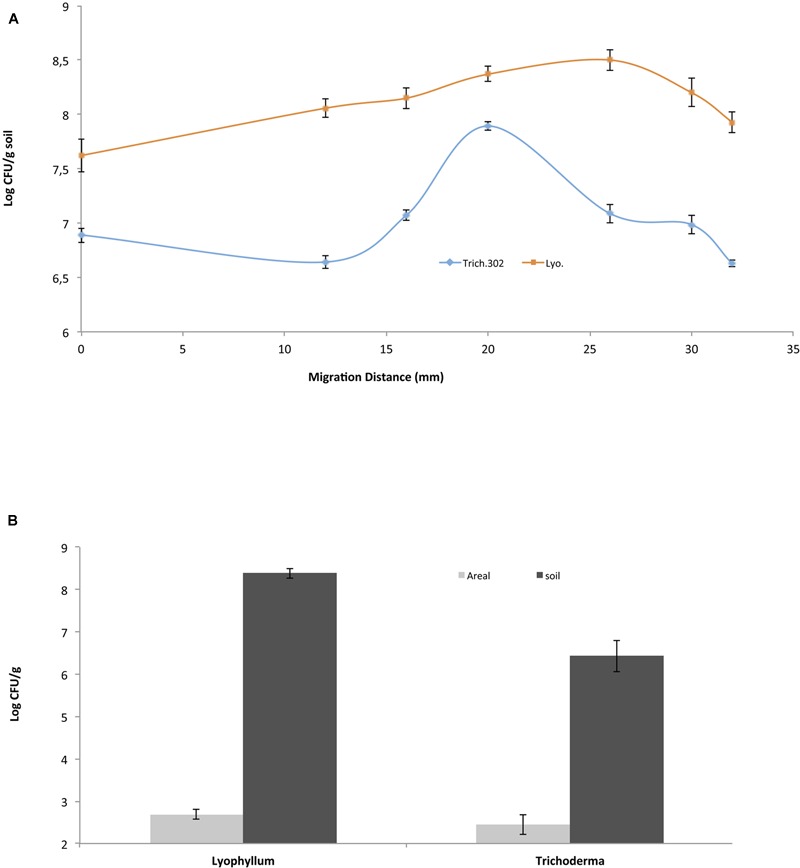
***Burkholderia terrae* BS001 interaction with two soil fungi-Microcosm experiments.** *B. terrae* BS001 distribution along fungal hyphae **(A)** at different spatial points sampled on the same day for *Lyophyllum* sp. strain Karsten (Lyo; represented by squares) and *Trichoderma asperellum* 302 (Trich302; represented by diamonds) and **(B)** on different hyphal types, i.e., aboveground (gray) or belowground (black).

To evaluate the spatial distribution of BS001 cells on the total (soil-bound and aerial) hyphae of *Lyophyllum* sp. strain Karsten and *Trichoderma asperellum* 302, different sites along the distal length of growth were sampled on the same day (day 15 after inoculation) from the soil microcosms (**Figure 1A**). First, the control microcosms did not show any bacterial migration, confirming previous data (Nazir et al., 2014). In the fungal-containing microcosms, the BS001 cell numbers at the inoculation site were similar for both fungi, at about 10^7^ CFU per g soil. These then increased and decreased differentially toward the distal ends, i.e., the hyphal fronts (**Figure 1**). As shown in this Figure, *Lyophyllum* sp. strain Karsten supported relatively consistent bacterial loads on the hyphae, while *Trichoderma asperellum* 302 showed an increase in bacterial numbers, followed by a slight decrease. The microscopic observations confirmed these observations.

#### Colonization of Soil-Bound vs. Aerial *Lyophyllum* sp. Strain Karsten Hyphae by *B. terrae* BS001 Cells

In a second analysis, the bacterial loads on soil-bound versus aerial hyphae were studied in the *Lyophyllum* sp. strain Karsten and *Trichoderma asperellum* 302 microcosms. From the samples, aerial hyphae were scraped off with sterilized forceps, whereas the remaining soil beneath the aerial hyphae served to sample the soil-bound hyphae. The distribution of fungal hyphae (m per g soil) over the below- and aboveground compartments was 6.8 (0.8 [standard deviation])/53.0 (0.7) for *Lyophyllum* sp. strain Karsten and 16.6 (5.8)/29.0 (11.5) for *Trichoderma asperellum* 302, respectively. The bacterial loads on the former hyphae of *Lyophyllum* sp. strain Karsten were about 10^8^ CFU per g of dry soil (estimated to equal 0.15 × 10^8^ CFU per m of hyphal tissue, whereas those on the aerial ones were <1/m of hyphal tissue (∼500 CFU per g), as also confirmed by microscopic observations. Thus, the *B. terrae* BS001 CFU numbers on the soil-dwelling hyphae were significantly (orders of magnitude) higher than those on the aerial hyphae (*P* < 0.05) (**Figure 1B**). Similar observations were made with *B. terrae* BS001 migrating along with *Trichoderma asperellum* 302. In the latter experiment, the estimated bacterial loads on the soil-dwelling versus the aerial hyphae were about 0.2 × 10^6^ per m and <1/m (∼300 CFU per g), respectively. These values were also significantly different from each other (*P* < 0.05).

### *B. terrae* BS001 Interaction with *Lyophyllum* sp. Strain Karsten in Microtiter Plates

#### *Lyophyllum* sp. Strain Karsten Growth

To elucidate if *Lyophyllum* sp. strain Karsten was able to adhere to, and grow in, the wells of a polystyrene microtiter plate, fungal growth was monitored over 72 h. The *Lyophyllum* sp. strain Karsten mycelium rapidly adhered to the surface of the wells and started to develop robust biomass after 3 h, which grew continuously further until 72 h (Supplementary Figure S2). In the light of the data indicating rapid growth, a 1.5 h adherence period was used in the ensuing fungal-bacterial interaction experiments. In these, observations were made by CLSM and SEM.

#### CLSM Observation of the *B. terrae* BS001-GFP -*Lyophyllum* sp. Strain Karsten Interaction

**FIGURE 2:**
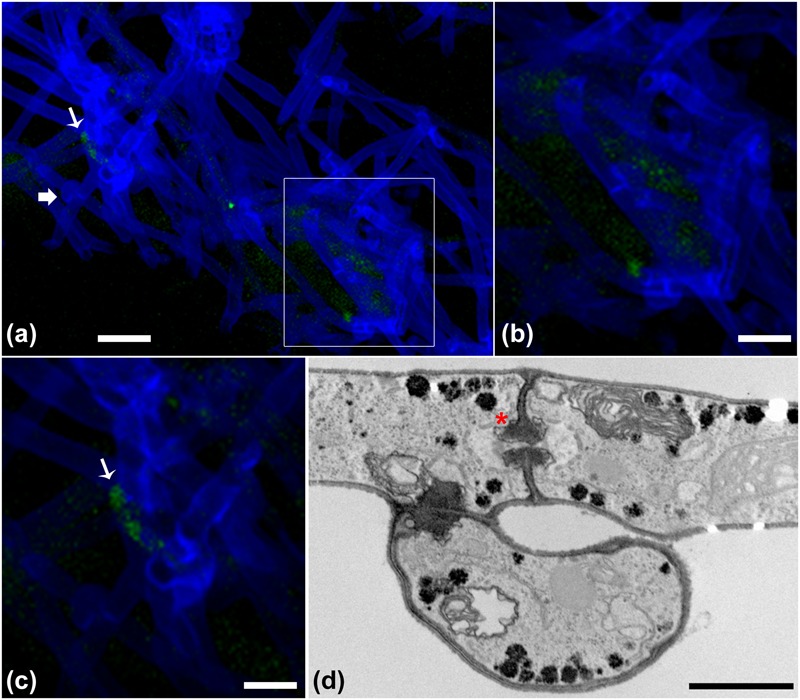
**Microscopy of the interaction between *B. terrae* BS001-GFP and *Lyophyllum* sp. strain Karsten. (a-c)** Green *B. terrae* BS001 cells attached to *Lyophyllum* sp. strain Karsten hyphae (blue). Cells cluster in specific hyphal regions, coined hot spots. Magnification 63×, bar: 20 μM. **(b)** Magnified image of a hot spot area (indicated with a white square). **(c)** Virtual zoom of a hot spot area indicated with a white narrow arrow. Calcofluor white staining showed ‘*clamps’* (bold white arrow), which are unique reproductive structures of *Basidiomycetes*. **(d)** Transmission electron microscopy (TEM) of *Lyophyllum* sp. strain Karsten hyphae, showing a characteristic dolipore pore (red ^∗^) and a *clamp*, formed during asexual reproduction. Bar: 1 μm.

The progression of the *B. terrae* BS001-GFP -*Lyophyllum* sp. strain Karsten interaction was analyzed using CLSM. Reconstructed 3-D images clearly indicated that the *B. terrae* BS001-GFP cells adhered irregularly to the hyphal surfaces (**Figure 2**). The cells appeared to cluster in specific regions of the hyphae (white arrow in **Figures 2a,c**, higher magnification in **Figure 2b**). Calcofluor white proved to be an excellent marker that stained the *Lyophyllum* sp. strain Karsten cell wall, which ensured the perfect observation of the complex network of hyphae that composes the fungal mycelium (**Figure 2**). We also observed an important sexual reproduction structure, called the “*clamp”* (**Figure 2a**, white bold arrow).

#### Electron Microscopic Observation of the *B. terrae* BS001 -*Lyophyllum* sp. Strain Karsten Interaction

**FIGURE 3:**
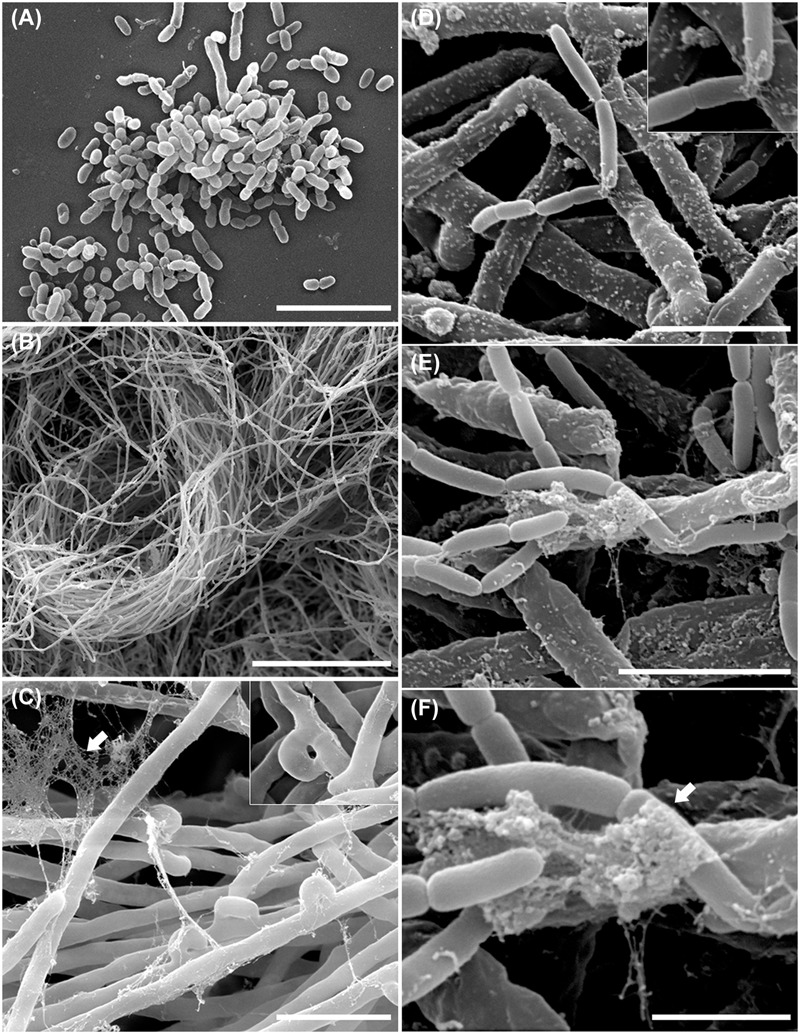
**Ultrastructural study of the *B. terrae* BS001 interaction with *Lyophyllum* sp. strain Karsten.** Scanning electron microscopy (SEM) images of **(A)** *B. terrae* BS001; **(B-C)** Mycelia of *Lyophyllum* sp. strain Karsten, showing many “*clamps*” (inset) and extracellular material (white arrow); **(D-F)** Bacterial-fungal interactomes showing *B. terrae* BS001 cells enfolded by extracellular material (white arrow). Bars: 50 μm **(B)**; 5 μm **(A,C)**; 2,5 μm **(D,E)**, and 1 μm **(F)**.

In order to examine the *B. terrae* BS001 -*Lyophyllum* sp. strain Karsten interaction at higher resolution, SEM was applied to systems that had co-existed for 1.5 h and then had been thoroughly washed. Controls included fungus-only and bacterium-only samples. Clearly, the controls showed that both the bacterial cells (**Figure 3A**) and the hyphal networks (**Figure 3B**) were well preserved. These observations also confirmed the abundant presence of *clamps* in the fungal networks (**Figure 3C**, inset). The latter were better visualized by TEM (**Figure 2D**), confirming the intense asexual reproduction inside the mycelia. Furthermore, large amounts of extracellular material adhering to the *Lyophyllum* sp. strain Karsten hyphae, also connecting these to each other, were observed (**Figure 3C**). Next, analyses of the interactomes showed the *B. terrae* BS001 cells to adhere to the fungal hyphae in an irregular way (**Figures 3D**-F). Often, these cells were found to be enfolded in extracellular material (**Figures 3D**-F, insets). The electron microscopic observations thus confirmed that the strain BS001 cells formed clusters at particular “hot spot” points of interaction with the fungal hyphae (**Figures 3E,F**). We surmised that such points of interaction might be characterized by particular fungal cell surface compounds that served as anchoring points for the bacterial cells.

### Structural Analysis of *Lyophyllum* sp. Strain Karsten CMHs

In a recent paper, we provide the arguments for a focus on CMHs as dynamic molecular ‘rafts’ that can become surface-exposed in fungal hyphae (Haq et al., 2016). We thus questioned the putative role of such CMH ‘anchors’ as sites for attachment of mycosphere bacteria. Here, we produced CMH from *Lyophyllum* sp. strain Karsten as shown in Supplementary Figure S1. After extraction with mixtures of chloroform and methanol followed by different chromatographic separation steps, an orcinol-reactive band was detected by HPTLC, with a chromatographic mobility corresponding to a standard bovine brain cerebroside.

**FIGURE 4:**
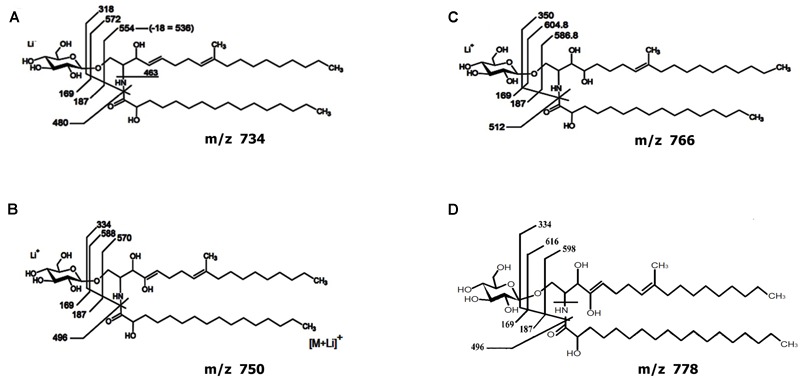
**Proposed structures for the CMH species of *Lyophyllum* sp. strain Karsten.** Structures composed by a hexose, a long chain base, 9-methyl-4,8-sphingadienine **(A)** or 4-OH-9-methyl-4,8-sphingadienine **(B–D)** and hydroxylated C_16_:0 fatty acid **(A–C)** or a hydroxylated C_18_:0 fatty acid **(D)**.

Analysis of the GlcCer-enriched fraction by positive ion mode ESI-MS then showed a cluster of singly charged ions with m/z ranging from 730 to 800 (Supplementary Figure S3), with a profile similar to that of other fungal cerebrosides (Barreto-Bergter et al., 2011). Four major ion species were observed at m/z 734, 750, 766 and 778. These were subjected to ESI-MS/MS analysis (Supplementary Figure S3). The loss of 162 units, common to all CMH analyzed and diagnostic of a monosaccharide unit, gave rise to major daughter ions at m/z 574, 588, 604 and 616 [M – hexose + Li^+^], corresponding to the ceramide monolithiated ion from the parental ions at m/z 734, 750, 766 and 778, respectively. The daughter ion at m/z 480 is consistent with the loss of an OH-C_16_ fatty acid (Supplementary Figure S3). Two fragments at m/z 187 and 169 confirmed the presence of a hexose. The difference of 16 units observed among CMH structures (Supplementary Figures S3B,C) remained detectable after loss of either monosaccharide or fatty acid, suggesting that the long chain base could have an extra hydroxyl group. The daughter ion at m/z 496 is consistent with the loss of a OH-C_18_ fatty acid present in the ceramide from structures B and D (**Figure 4**). Based on these observations, we concluded that the glycosphingolipid structures (**Figure 4**) consist of a hexose, a long chain base, 9-methyl-4,8-sphingadienine (**Figure 4A**) or 4-OH-9-methyl-4,8-sphingadienine (**Figures 4B–D**) and hydroxylated C_16_:0 fatty acid (**Figures 4A–C**) or a hydroxylated C_18_:0 fatty acid (**Figure 4D**).

### Is the Adherence of *B. terrae* BS001 Cells to *Lyophyllum* sp. Strain Karsten Hyphae Aided by a Glycosphingolipid?

**FIGURE 5:**
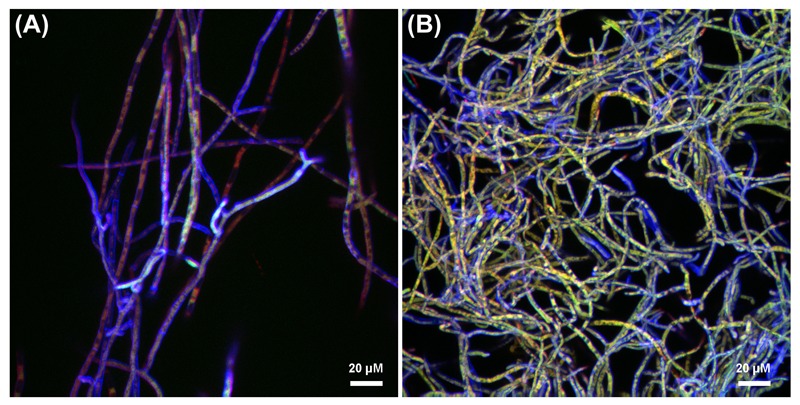
**Confocal laser scanning microscopy (CLSM) reconstructed Z-stack images of *Lyophyllum* sp. strain Karsten interaction with *B. terrae* BS001.** Magnification 63×, bar: 20 μM. The fungal cell wall was stained with Calcofluor white M2R (blue fluorescence), *B. terrae* BS001-GFP is visible by green fluorescence and the antibodies binding to CMH on the fungal cell wall were conjugated to alexafluor-546 dye (red fluorescence). **(A)** Three hours of interaction; **(B)** 24 h of interaction.

In order to elucidate whether CMH participates in the *Lyophyllum* sp. strain Karsten interaction with *B. terrae* BS001, we combined visual and quantitative analyses, using CLSM. Initially, the interaction was evaluated at two different times. The results showed that, after 3 h, in the treatment without anti-fungal antibodies, a considerable amount of bacterial cells had adhered to the fungal hyphae (**Figure 5A**). After 24 h, as both fungi and bacteria had grown, the fungal biomass had increased and new bacterial cells had also attached to the hyphae (**Figure 5B**).

In the systems to which anti-CMH antibodies had been added before the interaction with bacterial cells, varied red, green, and red-green areas appeared. First, areas where red fluorescence overwhelmed the green were taken to indicate inhibition of bacterial adhesion by the antibody. Remarkably, other areas, with overlapping red-green fluorescence, indicated bacterial attachment next to that of antibody. Finally, areas where green fluorescence dominated indicated bacterial cell adherence at the expense of adherence of, and possible blockage by, the antibody. **Figure 5A** (3 h of interaction) shows areas in which the presence of the antibody appears to inhibit bacterial cell adherence (red > green fluorescence). In contrast, at 24 h, bacterial adhesion was observed to occur throughout most of the hyphae (**Figure 5B**). Further quantitative analyses were performed using the 3h interaction images.

**FIGURE 6:**
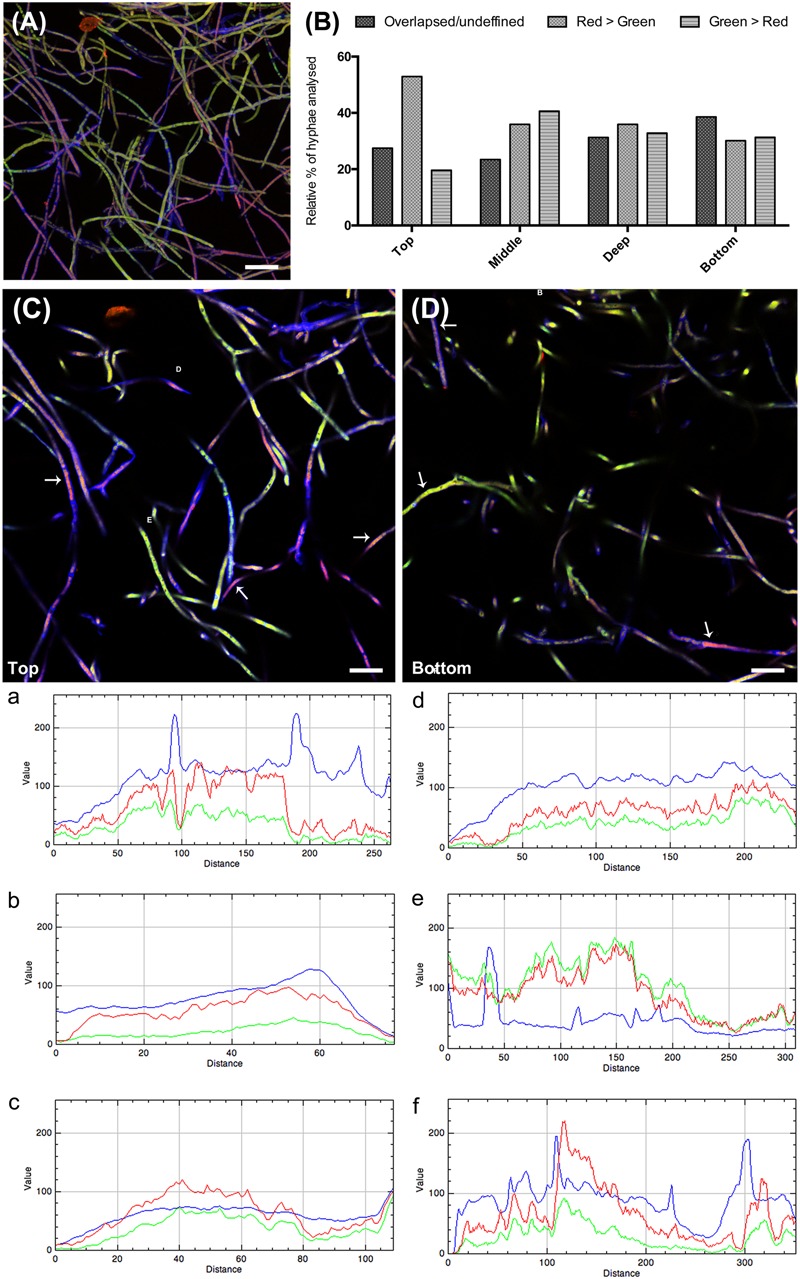
**The role of CMH in the *B. terrae* BS001 interaction with *Lyophyllum* sp. strain Karsten.** Magnification 63×, bar: 20 μM. The fungal cell wall was stained with Calcofluor white M2R (blue fluorescence), *B. terrae* BS001-GFP is visible by green fluorescence and CMH on fungal cell wall was stained with alexa-546 (red fluorescence, see legend **Figure 5**). **(A)** Z-stack reconstructed area after 3 h of *Lyophyllum* sp. strain Karsten and *B. terrae* BS001 interaction.**(B)** Quantitative analysis of fluorescence intensity, hyphal area in depth (of the biomass). As CMH antibodies were added before the interaction, red-green fluorescence intensity indicates areas where cell attachment to the fungal cell wall occurs via CMH. Areas with overlapsed red-green fluorescence indicate attachment through the antibody, areas with prevailing red fluorescence indicate inhibition of cell adhesion; areas where green fluorescence prevails indicate bacterial adhesion irrespective of antibody presence. **(C)** Top layer; **(D)** Bottom layer. Areas a, b, and c of image **(C)** and d, e, and f of image **(D)** were quantified regarding fluorescence intensity. Each graph: intensity of each fluorescent dye along the selected hyphae. This analysis showed that pre-incubation with CMH antibodies blocked cell adherence to the hyphal top layers more effectively than that to the bottom layers, where bacterial attachment occurred regardless of antibody presence.

Visual evaluation of the reconstructed Z-stack of all confocal planes at 3 h that were scanned showed two main patterns of bacterial adherence: (1) some hyphal areas were highly covered with green (gfp-marked) bacteria, while other areas had just few bacterial cells adhered. The latter were highly covered with anti-CMH antibody (**Figure 6A**). Analysis of the different layers of the biomass separately, as single confocal planes, revealed that the presence of anti-CMH antibodies inhibited cellular adherence to the fungal hyphae mostly in the top and middle layers. In contrast, in the deeper layers bacterial adherence was more evident along the hyphae (**Figure 6B**). A quantitative analysis of the fluorescence intensity of 40-50 hyphae/field for each fluorescent dye confirmed this contention. Representative fields of both the top and bottom layers are shown in **Figures 6C,D**, respectively. Three different representative areas of these images were selected and the graphs (a, b, c and d, e, f) show the fluorescence intensity patterns for each dye along the selected area. In the top layer, all three graphs show intense red fluorescence overwhelming the green color, indicating that hyphae covered with anti-CMH antibody have few (a, c) or no (b) adhering bacterial cells. Thus CMH molecules are indicated to be involved in the attachment of *B. terrae* BS001 to *Lyophyllum* sp. strain Karsten. In the bottom layers, the number of hyphae with intense green fluorescence surpassed the red fluorescence increases (**Figure 6D**, representative graph e), which indicated that the CMH molecules may not be the only moieties involved in bacterial attachment.

## Discussion

*Burkholderia terrae*, exemplified by strain BS001, has been reported to be enriched in the mycospheres of the ectomycorrhizal fungus *Laccaria proxima* and the basidiomycete *Lyophyllum* sp. strain Karsten in soil (Warmink and van Elsas, 2008; Warmink and van Elsas, 2009). The association with the mycelial networks in soil is important for migration over longer distances (mm range), as it facilitates the passage of air-filled voids in the soil (De Boer et al., 2005) and allows to access new soil microhabitats (Kohlmeier et al., 2005; Furuno et al., 2010; Nazir et al., 2010).

Nazir et al. (2014) indicated that strain BS001 has a ‘broad’ fungal-interactive ability, which can result in the emergence of bacterial cell agglomerates/biofilms associated with hyphal networks. Given this capacity, we surmised that physical parameters of the fungal surfaces determine, to a great extent, their colonization and subsequent migration success (Kohlmeier et al., 2005). Thus, fungal surfaces may provide shared local conditions that are conducive to colonization by the bacterial associate. Here, we examined the *B. terrae* BS001 – *Lyophyllum* sp. strain Karsten interactome with respect to discernable characteristics of the colonization process, including the hydrophobicity of the hyphae in response to the environment (as related to ‘conditions’), its ultrastructural aspects and the potential presence of a receptor moiety at the fungal surface.

In soil, for both *Lyophyllum* sp. strain Karsten and *Trichoderma asperellum* 302, the abundance of strain BS001 CFUs was by far the highest on the soil-bound hyphae (soil at about 70% of water-holding capacity, at which pores with neck sizes of 4 μm are estimated to be water-filled) as compared to the aerial hyphae. Hyphae growing in humid conditions such as in the soil were found to be more hydrophilic than aerial hyphae (**Table 1**). The more efficient colonization of the hydrophilic hyphae can be best explained by the presence of broader liquid films (Kohlmeier et al., 2005) which serve as dispersal and growth habitats for strain BS001, which itself was found to be hydrophilic (CA = 22 ± 1°). This underpins the importance of the compatibility of (micro-)habitats for growth and ecophysiological responses. The physicochemical surface properties of the hyphae may thus constitute important criteria that modulate the interactions of *B. terrae* BS001 with *Lyophyllum* sp. strain Karsten.

From the experiment in the microtiter plates, we concluded that *B. terrae* BS001 adheres to, and creates a biofilm around, the *Lyophyllum* sp. strain Karsten hyphae. Material, presumably consisting of extracellular polysaccharides (EPSs), was found around the BS001 cells. Such material was also found in the system with the fungus only. Hence, EPSs can be produced by *Lyophyllum* sp. strain Karsten hyphae and/or by the fungal-bacterial interacting pair under the conditions applied. The EPS may serve as a protectant as well as an anchoring medium for the bacterium, allowing it to move from one point to another. Furthermore, the matrix formed by the EPS may electrostatically interact with the charged surface of the fungus, establishing a physicochemical bond.

The ultrastructural study of the *Lyophyllum* sp. strain Karsten – *B. terrae* BS001 interactome confirmed that the bacterial cells form clusters at particular “points of interaction” with the fungal hyphae. Such cells not only adhered to the fungal surface, but were also enfolded in the extracellular material, confirming that they were organized in structures that resemble biofilms along the hyphae. This material, often connecting the hyphae, was also observed in *Lyophyllum* sp. Karsten preparations, suggesting it forms its own ‘biofilm’ *in vitro*. The same observation was recently made for other filamentous fungi like *A*sp*ergillus* sp. (Kaur and Singh, 2014) and *Mucorales* (Singh et al., 2011), and even multi-layered biofilms have been found (Sardi et al., 2014). The biofilm ‘mode of growth’ is also prevalent in bacteria in natural settings (Costerton et al., 1995), and the presence of an extracellular matrix (ECM) is key to biofilm success (Donlan and Costerton, 2002). It is associated with an increased resistance to stressors such as UV radiation, drought, phagocytosis and antimicrobial/pesticide/toxic compounds (Flemming and Wingender, 2010). In this study, we found a potential ‘coalescence’ of bacterial and fungal biofilms. Multi-species biofilms can prevail in nature (Hogan and Kolter, 2002; Brandl et al., 2011; Morales et al., 2013; Trejo-Hernández et al., 2014) if they confer a survival strategy to the partners. In such biofilms, the ECM, composed of secreted compounds, establishes a complex matrix structure (Flemming and Wingender, 2010). Here, polysaccharides secreted by *B. terrae* BS001 and *Lyophyllum* sp. strain Karsten cells may have incited the mixed biofilm, with implications for the life of both partners. Further studies should focus on the composition of this ECM and its role in bacterial adherence, co-migration and other interactive actions.

Given the fact that bacterial adherence occurred in clusters and was not spread along the entire hyphal network, we examined whether the adherence of BS001 cells to the fungal cell wall was mediated by a specific ligand-receptor system. From work with other fungi, it is known that, next to the common fungal cell wall compounds chitin and β-glucan, CMH moieties provide specific exposed sites at the fungal surface. These glycosphingolipids have been indicated as adhesion receptors for several medically related bacteria. Zhang and Zhang (1994), using a TLC overlay assay, identified sulfated glycolipids as the receptors for attachment of *Mycoplasma hyopneumoniae* to porcine cells. Moreover, *Borrelia burgdorferi* cells were found to bind specifically to galactosylceramide (GalCer) and glucosylceramide (GlcCer) present in various types of cells (Kaneda et al., 1997).

More recently, a specific sugar sequence, such as NeuAcα2→3 Galβ- (present as non-reducing end of the glycolipid) was found to function as the attachment site for *Vibrio harveyi* at the epithelial cells of fish intestines (Chisada et al., 2013). Concerning the binding of fungi such as *Cryptococcus neoformans, Candida albicans, Histoplasma capsulatum*, and *Sporothrix schenckii*, these were found to bind to lactosyl ceramide. Terminal galactose in the lactosyl ceramide was essential for the binding, as removal of this sugar abolished it (Jimenez-Lucho et al., 1990).

In order to elucidate whether CMH participates in the *Lyophyllum* sp. strain Karsten interaction with *B. terrae* BS001, we combined visual and quantitative analysis of CLSM Z-stacks. Clearly, in the top and middle layers of the interactome, blocking of CMH by an antibody hampered bacterial adherence, while in deeper layers strong bacterial adherence was evident along the hyphae. The collective results indicate that the fungal glycosphingolipid CMH can act as a receptor in the fungal cell wall for the attachment of *B. terrae* BS001 cells. However, the data also indicated that CMH might be part of a bigger structure that governs the physical interactions between *B. terrae* BS001 cells and the fungal hyphae.

In summary, our work confirmed that *B. terrae* BS001 colonizes the hydrophilic rather than the hydrophobic hyphae of *Lyophyllum* sp. strain Karsten, which highlights the relevance of local (soil) conditions, such as water availability, for the interactions. For instance, the aerial hyphae growing over the soil, being abundant, were very poor carriers of the bacterial cells, whereas those growing through the soil, being a relative minority, represented excellent colonization sites. We also showed that bacterial adherence occurs in specific areas of the hyphal surface of *Lyophyllum* sp. strain Karsten and that the fungal glycosphingolipid CMH can act as a receptor in the fungal cell wall for attachment of *B. terrae* BS001 cells. From our further studies, we then obtained evidence for the contention that *B. terrae* BS001 adheres to, and creates biofilms around, the *Lyophyllum* sp. strain Karsten hyphae, which also grows in a biofilm-related way. We hypothesized that, when together, *Lyophyllum* sp. strain Karsten and *B. terrae* BS001 form a mixed biofilm, in which the ECM also contributes to the interaction between the species.

## Author Contributions

TV, RN, GdS, RC, and LW conceived of and analyzed all experiments; TV, RN, SR, EB-B, LW, and JDvE analyzed the data, drafted, and revised the manuscript. All authors read and approved the final version to be published.

## Conflict of Interest Statement

The authors declare that the research was conducted in the absence of any commercial or financial relationships that could be construed as a potential conflict of interest.
