# Supplementary figures and images for: The Role of Hydrophobicity and Surface Receptors at Hyphae of Lyophyllum sp. Strain Karsten in the Interaction with Burkholderia terrae BS001 – Implications for Interactions in Soil
Source: Front Microbiol. 2016 Oct 27;7:1689. doi: 10.3389/fmicb.2016.01689 (PMC5081359; doi:10.3389/fmicb.2016.01689)

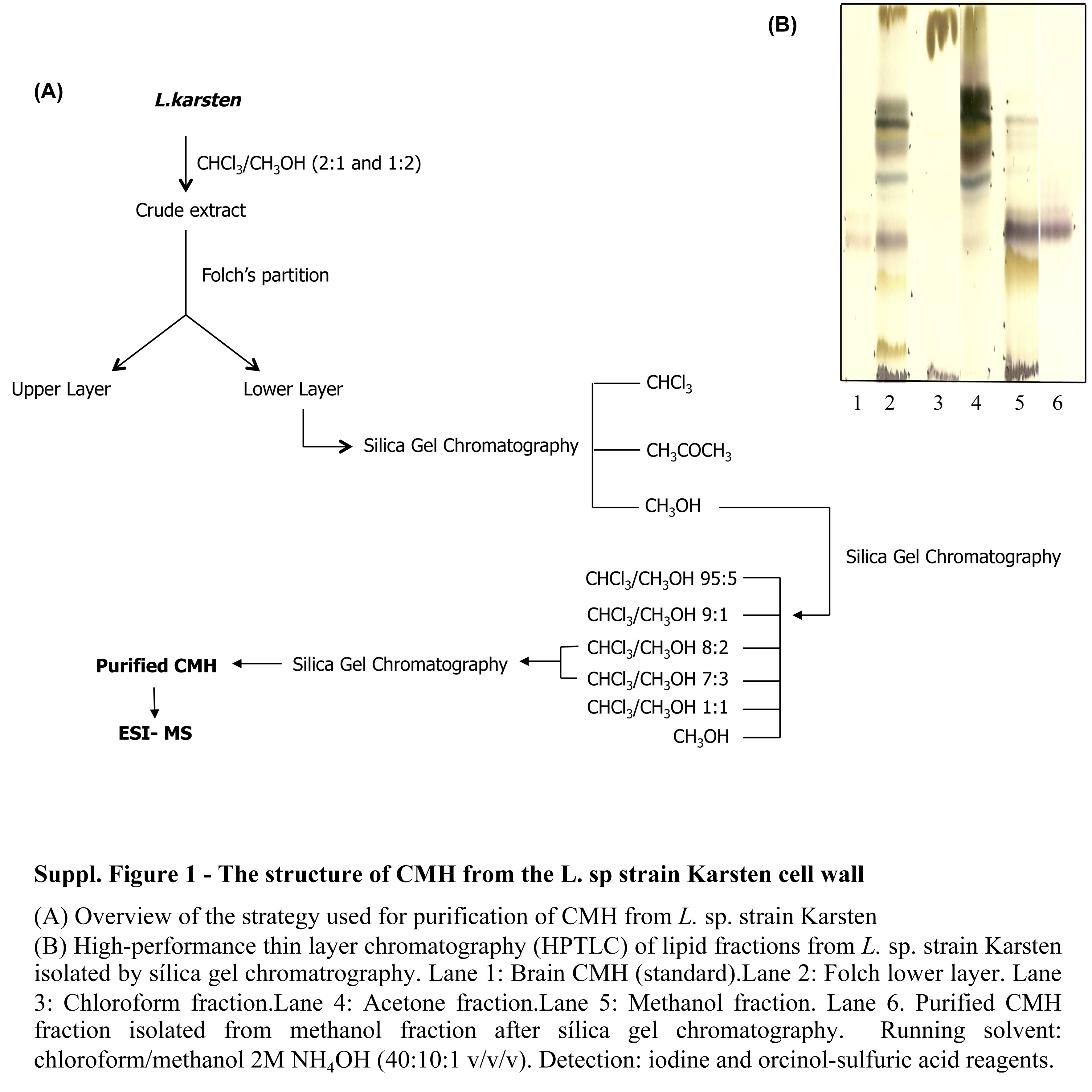

Supplement: Supplementary file 1 [file Image_1.TIF]

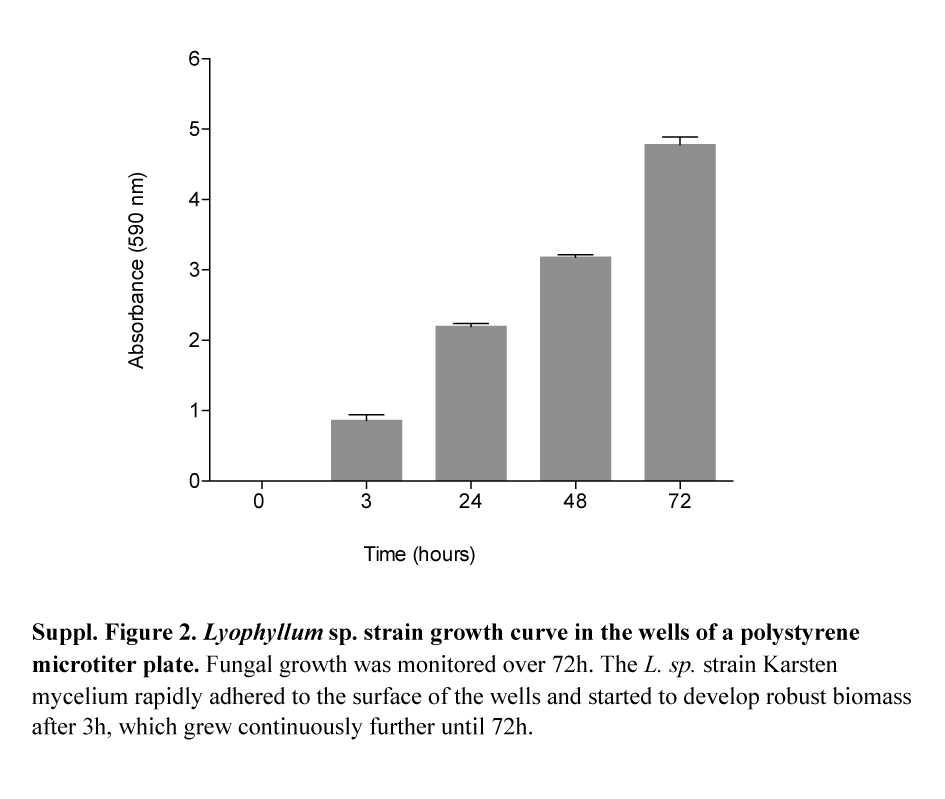

Supplement: Supplementary file 2 [file Image_2.TIF]

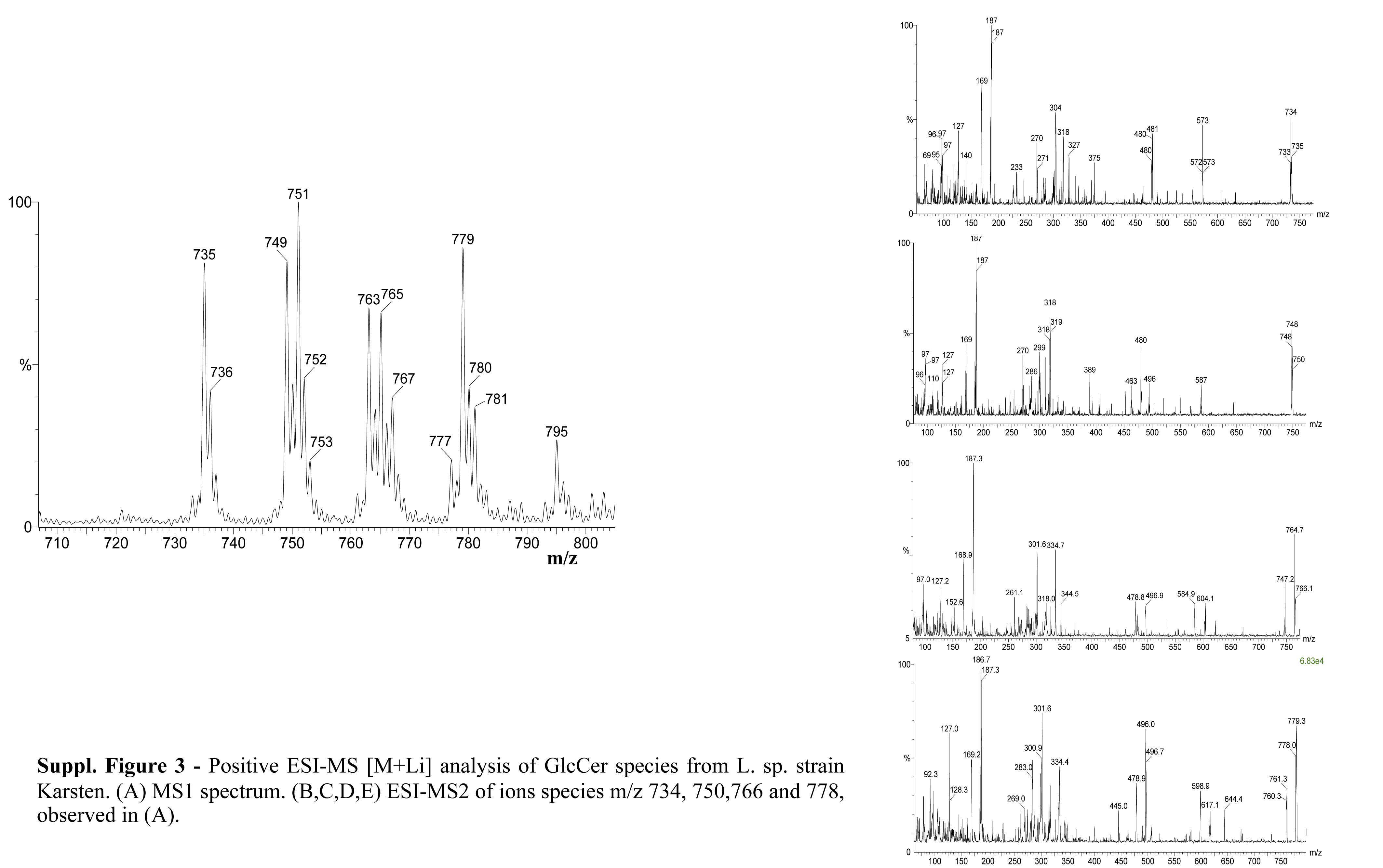

Supplement: Supplementary file 3 [file Image_3.TIF]
